# In Silico Prediction of Anti-Infective and Cell-Penetrating Peptides from *Thalassophryne nattereri* Natterin Toxins

**DOI:** 10.3390/ph15091141

**Published:** 2022-09-13

**Authors:** Gabrielle Lupeti De Cena, Bruna Vitória Scavassa, Katia Conceição

**Affiliations:** Laboratory of Peptide Biochemistry, Universidade Federal de São Paulo (UNIFESP), São José dos Campos 12231-280, Brazil

**Keywords:** bioactive peptides, antimicrobial peptides, cell-penetrating peptides, in silico prediction, ADMET, hydrophobicity

## Abstract

The therapeutic potential of venom-derived peptides, such as bioactive peptides (BAPs), is determined by specificity, stability, and pharmacokinetics properties. BAPs, including anti-infective or antimicrobial peptides (AMPs) and cell-penetrating peptides (CPPs), share several physicochemical characteristics and are potential alternatives to antibiotic-based therapies and drug delivery systems, respectively. This study used in silico methods to predict AMPs and CPPs derived from natterins from the venomous fish *Thalassophryne nattereri*. Fifty-seven BAPs (19 AMPs, 8 CPPs, and 30 AMPs/CPPs) were identified using the web servers CAMP, AMPA, AmpGram, C2Pred, and CellPPD. The physicochemical properties were analyzed using ProtParam, PepCalc, and DispHred tools. The membrane-binding potential and cellular location of each peptide were analyzed using the Boman index by APD3, and TMHMM web servers. All CPPs and two AMPs showed high membrane-binding potential. Fifty-four peptides were located in the plasma membrane. Peptide immunogenicity, toxicity, allergenicity, and ADMET parameters were evaluated using several web servers. Sixteen antiviral peptides and 37 anticancer peptides were predicted using the web servers Meta-iAVP and ACPred. Secondary structures and helical wheel projections were predicted using the PEP-FOLD3 and Heliquest web servers. Fifteen peptides are potential lead compounds and were selected to be further synthesized and tested experimentally in vitro to validate the in silico screening. The use of computer-aided design for predicting peptide structure and activity is fast and cost-effective and facilitates the design of potent therapeutic peptides. The results demonstrate that toxins form a natural biotechnological platform in drug discovery, and the presence of CPP and AMP sequences in toxin families opens new possibilities in toxin biochemistry research.

## 1. Introduction

Animal venoms contain a diverse and complex mixture of bioactive compounds that target various receptors to support the survival of venomous animals [1]. Several drugs derived from animal venoms have been approved by the FDA for human use, while other drugs are in clinical trials [2,3]. Recent advances in genomics and proteomics have improved the biochemical analysis of venoms [4]. The ability to rapidly screen venom compounds using high-throughput technologies and the prediction of new molecules encoded in toxins allows for harnessing the therapeutic potential of animal venoms.

Peptides are key role molecules found in all organisms and play a crucial role in many biological processes [5,6,7,8]. The large distribution and functional diversity of peptides increase their therapeutic potential [9,10,11]. Venom-derived peptides involved in defense and predation have long been exploited for medicinal, agricultural, and biotechnological applications [1,12]. Most of these peptides originate from a limited number of taxa of venomous terrestrial animals. However, several bioactive compounds from fish venoms have been isolated and characterized [13]. Prediction of new bioactive peptides (BAPs) derived from natterins from the venomous fish *Thalassophryne nattereri* by in silico analysis is the aim of this study.

*T. nattereri* is responsible for cases of envenomation of fishermen and bathers in the north and northeast of Brazil [14,15]. The most common sites of envenomation are the palm of the hands or soles of the feet [16]. The natterin family of toxins contains five orthologs: natterin 1–4 and -P [17]. Natterins are tissue-kallikrein-like enzymes and aerolysin-like pore-forming toxins responsible for the main toxic effects of *T. nattereri* venom: local edema, excruciating pain, and necrosis [18,19,20]. The degree of amino acid homology between natterin 1 and 2 is 84%, and these orthologs have 40% identity with natterin 3 and 4 (Figure S1). Natterin P is the shortest ortholog (71 amino acids) and shows 84% identity with the first 55 amino acid residues in the N-terminus of natterin 4 [17,20]. We hypothesize that natterins should be a source of BAPs with antimicrobial and cell-penetrating activity based on their pharmacological profile.

The therapeutic potential of venom-derived BAPs is determined by specificity, stability, and pharmacokinetic properties [21]. Two classes of BAPs—anti-infective or antimicrobial peptides (AMPs) and cell-penetrating peptides (CPPs)—share several physicochemical characteristics and are potential alternatives to antibiotic-based therapies and drug delivery systems, respectively. Since the plasma membrane selectively controls the transport of bioactive substances across cells, there is increased interest in developing novel strategies to overcome this barrier and increase bioavailability. In this context, peptide-based transport systems, such as CPPs, have come into focus, and their efficiency has been demonstrated in multiple applications [22,23,24,25].

AMPs are a large class of naturally occurring peptides with antibacterial and/or antifungal activity and can help overcome microbial resistance to conventional antibiotics [26,27,28]. Fusion of CPPs and AMPs produces multifunctional peptides capable of treating infections, cancer, obesity, and other diseases [29,30,31,32]. Thus, concerted efforts are being made to design new AMPs or CPPs [33,34,35,36,37]. Nonetheless, these BAPs have failed clinical trials, underscoring the need to optimize these peptides. In this context, the computer-aided design of BAPs has generated crucial information on the physicochemical characteristics and biological activities of BAPs, allowing analyzing these proprieties and activities before peptide synthesis. Several methods have been developed to predict AMPs and CPPs and evaluate physicochemical properties [38,39]. 

AMPs and CPPs can be derived from known protein sequences. However, analyzing the physicochemical properties of proteins using experimental techniques is expensive and laborious. In silico approaches are faster, cheaper, and less laborious, enabling the large-scale screening and identification of BAPs with application in biomedicine and pharmacology [40]. 

Several BAP prediction tools have been developed using different data features and machine learning methods [34,40,41], and the performance of these tools varies depending on these features and the nature of the training technique. Most prediction methods use single classifier models such as support vector machine (SVM), discriminant analysis, fuzzy k-nearest neighbor, and deep learning. Other methods use decision tree classifiers such as ensemble models and random forests [42]. 

In silico approaches have facilitated the design of highly effective engineered peptides with cell-penetrating, antimicrobial, and anticancer activity [43,44,45,46,47,48]. However, as peptides gain ground over small molecule drugs [2,49], some disadvantages must be overcome, including chemical and physical instability [50], high susceptibility to proteolytic degradation [51], short half-life and high clearance [52], slow tissue penetration [53], and high cytotoxicity [53]. In this context, machine learning techniques have been used to screen peptide template libraries based on physicochemical properties and absorption, distribution, metabolism, excretion, and toxicity (ADMET) parameters. This study evaluated the physicochemical and ADMET profiles of newly predicted peptides derived from natterins.

## 2. Results and Discussion

There has been an increased interest in therapeutic peptides as potential drug candidates [54]. Several studies identified and characterized a wide range of therapeutic peptides, including tumor-homing peptides [55], CPPs [56], AMPs [57], and anticancer peptides (ACPs) [58,59,60], and used these peptides for treating cancer, diabetes, and cardiovascular diseases. As a result of these efforts, several peptides have entered clinical trials over the past two decades [54]. Nonetheless, only a few peptide-based drugs are used clinically. Therefore, many research groups have focused on computational design based on physicochemical and structural features to produce potent and broad-spectrum peptides [9]. Several computational tools have been used to design peptide-based drug candidates [41]. This study predicted and characterized novel and potent BAPs derived from *T. nattereri* natterins by in silico analysis.

### 2.1. Identification of Potential Natterin-Derived AMPs and CPPs

Fifty-seven natterin-derived BAPs were identified using the web servers AMPA, CAMP, AmpGram, C2Pred, and CellPPD. These peptides were named according to the original sequence (natterin 1, 2, 3, 4, or P) and the order in which they were identified. For instance, the first peptide derived from natterin 1 was named NATT1_1, the second was named NATT1_2, etc. Some peptide sequences were homologous to more than one natterin. In these cases, the numbering of the source natterin was added to the nomenclature. For instance, the peptide RTYRGGKKTQTTTKGVYRTTQV was the first to be identified as belonging to natterin 1 and 2 and thus was named NATT1.2_1. 

All predicted AMPs and CPPs and their respective scores (SVM, RF, or artificial neural networks (ANNs)) and probability scores are listed in Table 1. Nineteen peptides were classified as AMPs, of which seven, three, six, and three belonged to natterin 1, 2, 3, and 4, respectively. Eight CPPs were found, of which one and seven belonged to natterin 2 and 4, respectively. Thirty sequences shared AMP and CPP characteristics, of which five, eleven, four, five, and five sequences belonged to natterin 1, 2, 3, 4, and P, respectively. In the C2Pred web server, peptides with scores of <0.5 and ≥0.5 are classified as non-CPPs and CPPs, respectively. Although some peptides were predicted to be CPPs by CellPPD, C2Pred classified them as non-CPPs. For instance, the natterin 3-derived peptide NATT3_10 was classified as CPP and non-CPP using CellPPD (SVM score of 0.1) and C2Pred (score of 0.48551), respectively.

The length of the predicted peptides varied from 10 to 23 amino acid residues. In the 1980s, most peptides entering clinical development were less than 10 amino acids long. However, the length of engineered peptides increased over the years due to improvements in chemical synthesis and manufacturing technologies [61,62]. In the current decade, candidate peptides have up to 40 amino acids, suggesting that length is no longer a limitation. Nonetheless, most drug candidates have 10 amino acid residues are still the majority for peptide drug development. In the present study, 63.8% of the peptides presented 10 amino acids.

### 2.2. Physicochemical Properties and Membrane-Binding Potential

**Figure 1:**
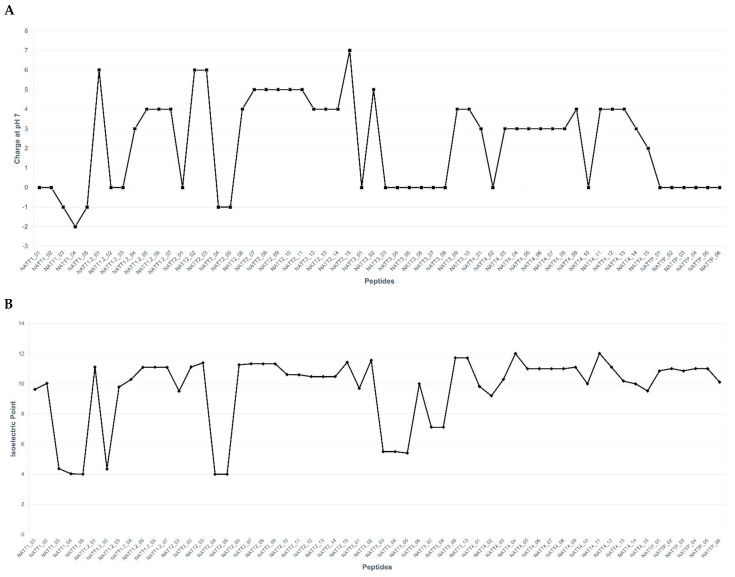
Graphical representation of the physicochemical properties of 57 peptides: (**A**) total net charge at pH 7.0 and (**B**) isoelectric point.

The following physicochemical characteristics were analyzed: net charge, pI, molecular weight (MW), amphipathicity, water solubility, hydrophobicity, hydrophobicity ratio, and charge. CPPs and AMPs are rich in particular amino acids, such as Arg, Trp, Pro, Gly, Cys, and His. The hallmark of these two classes of peptides is an abundance of basic (Arg and Lys) residues and/or Trp. The charge and pI values are shown in Figure 1. Forty-six (81%) peptides were cationic, nine (16%) were anionic, and two (3%) were neutral. The modes of action are determined by the physicochemical features of amino acid residues [63]. The net positive charge and amphipathicity significantly influence the bioactivity of AMPs and most CPPs [27]. The net positive charge affects initial electrostatic interactions with anionic phospholipids and lipopolysaccharides in the plasma membranes of certain pathogens [64]. In turn, mammalian cells, such as red blood cells, are composed primarily of zwitterionic phospholipids in the outer leaflet of their membranes, which are more strongly affected by peptide hydrophobicity than by positive charges [65]. Highly hemolytic peptides interact with phosphatidylcholine, an abundant component of zwitterionic membranes [66]. In contrast, cholesterol inhibits peptide binding in mammalian cell membranes [67].

In vitro and in vivo studies need controlled and accurate peptide concentration; hence, peptide solubilization is a critical step for successful assays. Consequently, poor peptide solubilization can introduce experimental errors and lead to experimental failure [21]. In this respect, the solubility of bioactive peptides depends on the molecular length and the number of hydrophobic amino acids (Table 2) [68]. Peptides with a high percentage (≥50%) of hydrophobic amino acids are generally partially soluble in aqueous solutions [20,69]. Our results showed that 22% of the peptides were stable (Table S1). The stability of drug candidates is critical for manufacturing the active pharmaceutical ingredient and for enabling formulation of a stable compound. Further, these properties enable producing peptides with different routes of administration, including topical, subcutaneous, and fast intravenous push preparations [70].

**Figure 2:**
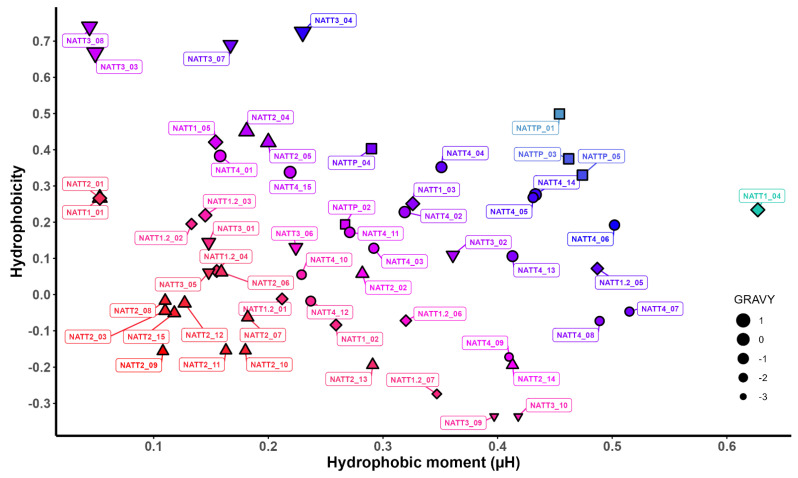
Hydrophobic plot and hydropathicity (GRAVY) of predicted natterin-derived antimicrobial and cell-penetrating peptides. The *X*-axis represents the helical hydrophobic moment (μH), and the *Y*-axis represents the corresponding hydrophobicity (H). The peptides in blue and red are the most and least internalized, respectively. NATT1_04 (green) had high cell internalization and cytotoxicity.

The hydrophobic properties for all peptides were calculated, and a plot representing hydrophobicity vs. hydrophobic moment vs. GRAVY of peptides allowed us to visualize the differences in terms of hydrophobicity between each peptide (Figure 2). The hydrophobic plot can indicate that diminution of hydrophobicity and amphipathicity of the natterin peptides decreases their cellular uptake and that the substantial increase in these parameters can lead to an increase in their cytotoxicity. This suggests that carefully controlling these parameters can enhance peptide internalization and that above this threshold value it is expected that unwanted toxicity starts to appear. The nature of hydrophobic residues, positioning, and aromaticity are harmful mainly to CPPs’ fate in terms of the reversibility of the membrane interaction and final membrane crossing. Studies with Trp-rich peptides revealed that less hydrophobic residues and more interfacial ones can contribute to the peptides establishing more transitory interactions with the membrane in part due to a less deep membrane insertion. This type of flexible membrane interaction is important to prevent the peptide from being locked in the membrane interior and to trigger translocation into membranes [71].

**Figure 3:**
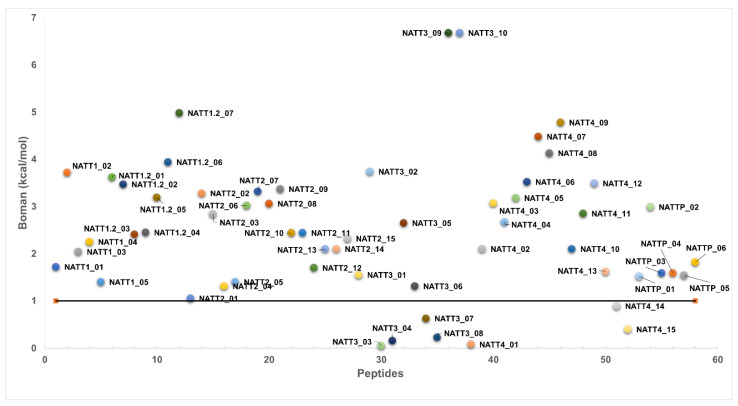
Boman indexes of predicted antimicrobial and cell-penetrating peptides.

The Boman index estimates protein-binding potential and is calculated on the basis of the cyclohexane-to-water partition coefficient of the respective amino acid side chains divided by the total number of amino acid residues within the peptide [72]. A high index (>2.48) indicates high binding potential (e.g., hormones), whereas a low index (≤1) indicates fewer side effects (e.g., lower toxicity to mammalian cells) [72]. Seven (12%) sequences had a Boman index below 1 (Figure 3). The sequences YVCSCGCSSG (NATT3_03) and LYVAKNKYGLGKL (NATT4_01) presented the best index (0.05 and 0.08, respectively) and will be further chemically synthesized and assayed in vitro and in vivo. These Boman values were expected as AMPs typically do not bind to other proteins but penetrate and disrupt the plasma membrane. Given the amphiphilic nature of CPPs, ACPs, and antiviral peptides (AVPs), strong interaction with and deep penetration into the anionic lipid bilayers are expected for BAPs, making the plasma membranes prone to disruption, endocytosis, and/or direct translocation [73,74]. The Boman index of our peptides ranged from 4.0 to 6.7, which is higher than the range reported previously (∼1.0–3.5) [73].

**Figure 4:**
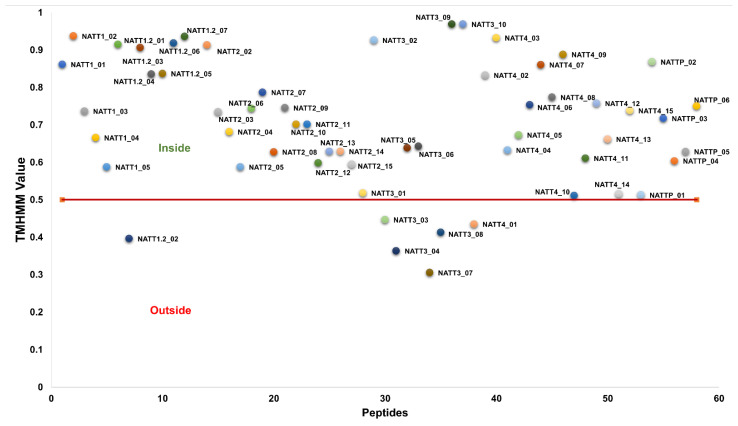
Cellular localization of predicted antimicrobial and cell-penetrating peptides using the TMHMM web server.

The cellular localization of each peptide was evaluated using the TMHMM server to estimate the probability of peptide translocation across lipid membranes. The results showed that 90% of the predicted peptides were located in the cell membrane. The membrane-binding potential and cellular localization of CPPs are shown in Figure 4 and Table S2. 

### 2.3. Prediction of Biological Activities

#### 2.3.1. Immunogenicity, Allergenicity, and Toxicity 

Immunogenicity assessment is a crucial step in the drug development process. The complexity of the immune system demands the use of multiple approaches to predict the immunogenicity of biopharmaceuticals. Experimental studies are straightforward, such as in vitro, in vivo, and ex vivo, but are sometimes expensive and time-consuming, and their results need to be confirmed [75]. Immunogenicity was analyzed using the Immune Epitope Database (IEDB), a database of epitopes and immune receptors [76] (Table 3). Higher scores indicated a higher probability of triggering an immune response. The immunogenicity of all predicted peptides was lower than 0.7, demonstrating that they did not cause immune responses [77,78].

Given the risk of inducing an immediate type I (IgE-mediated) allergic response, the allergenic potential of druggable proteins and peptides should be determined before they are marketed. The allergenic potential was evaluated using the AllerTOP web server by applying auto-cross covariance transformation to build a dataset of known allergens and developing alignment-independent models for allergen recognition based on the physicochemical properties of proteins [79]. The tool uses five machine learning methods for protein classification, including partial least squares discriminant analysis, logistic regression, decision tree, naïve Bayes, and k-nearest neighbors. In addition, AllerTOP attempts to identify the most likely route of exposure. AllerTOP outperforms other allergen prediction models, with a sensitivity of 94% [79]. Of the 57 predicted sequences, 35 were classified as non-allergenic (Table 3).

Toxicity was assessed using ToxinPred software [80,81], which uses the following datasets to train and test SVM models: (1) a main dataset (1805 toxin sequences from experimentally validated peptides/proteins (positive examples) and 3593 non-toxin sequences from SwissProt (negative examples)), (2) a main independent dataset (303 toxin sequences and 300 SwissProt non-toxin sequences), (3) an alternative dataset (1805 toxin sequences (positive examples) and 12,541 non-toxin sequences from TrEMBL (negative examples)), (4) and an alternative independent dataset (303 toxin sequences from SwissProt and 1000 non-toxin sequences from TrEMBL). All identified peptide sequences were classified as non-toxic (data not shown).

#### 2.3.2. Antiviral and Anticancer Potential

The control of viral diseases is challenging because of increased resistance to antiviral drugs and the emergence of new viral pathogens. AVPs, a subset of AMPs, are a potential source of therapeutics useful for preventing and treating viral infections [82]. The ability of AVPs to target various stages of the viral lifecycle, ranging from their attachment to host cells to their ability to impair viral replication within the cells, has been the subject of multiple studies [83,84,85]. Sixteen sequences were predicted to be AVPs, of which four had a score above 90%. NATT1.2_05 and NATT1.2_06 presented the highest scores (0.964 and 0.962, respectively). AVPpred predicts AVPs based on experimentally validated positive and negative datasets. 

Cell membrane properties differ between cancer cells and healthy cells [86]. For instance, the membrane fluidity of cancer cells is higher than that of healthy cells [87]. In addition, the membrane of cancer cells has a higher negative charge, larger surface area due to the higher number of microvilli, and higher fluidity than that of healthy cells. ACPs, a subset of AMPs, are toxic to cancer cells [86]. ACPs have 5–30 cationic amino acid residues that adopt an α-helical or β-sheet structure but can assume a linear structure [88,89]. In the present study, 37 peptides were predicted to be ACPs. The physicochemical properties of ACPs determine electrostatic interactions with the anionic cell membrane of cancer cells and thus allow the selective killing of these cells [90]. ACPs have several advantages over small molecule cancer drugs. For instance, the shorter half-life decreases the probability of resistance. Moreover, ACPs have low toxicity, high specificity, high solubility, and good tumor penetration ability, demonstrating their great potential in cancer therapy [88,89,90,91]. The half-life of the predicted ACPs in mammalian cells varied from 1 to 100 h (Table 3). Compared to biologics, peptides have a much shorter circulatory half-life (days vs. weeks), resulting in the need for sub-optimal frequent drug administration [92].

#### 2.3.3. Prediction of ADMET Properties

The analysis of biochemical processes from drug administration to elimination plays a crucial role in lead optimization. An ideal peptide drug should be quickly absorbed into the systemic circulation and eliminated without affecting pharmacological activity. Further, ideal candidates should be non-toxic. The analysis of ADMET parameters is essential in drug discovery. ADMET properties were predicted using the web server ADMETlab version 2.0 [93] (Table 4). The parameters analyzed were blood–brain barrier (BBB) penetration, Caco-2 permeability, volume of distribution (VD), plasma protein binding (PPB), human intestinal absorption (HIA), clearance (CL), half-life (T1/2), skin sensitization, AMES toxicity, carcinogenicity, and synthetic accessibility (SA) score (Table 4). All compounds had positive HIA, indicating the high ability to cross the intestinal barrier. Higher BBB penetration is associated with higher lipophilicity profiles and higher uptake. The calculated value for the BBB was shown to have a high likelihood of being negative. PPB is an important parameter in drug safety assessments since compounds with high PPB (>90%) have a narrow therapeutic index, whereas compounds with low PPB are considerably safer. All analyzed peptides had low PPB, indicating a good therapeutic index. Caco-2 cells, derived from human colon adenocarcinoma cells, have permeability functions similar to those of intestinal enterocytes and are used to predict intestinal drug absorption in vivo. All analyzed compounds had the best scores (greater than −6.47) in Caco-2 cell permeability assays. Regarding carcinogenicity, none of the analyzed peptides showed potential to cause cancer. The results of the AMES test showed that none of the peptides were genotoxic. The analysis of other toxicity parameters, such as hERG inhibition, hepatotoxicity, and skin sensitization, revealed that all peptides were safe. The SA score estimates the ease of synthesis (Table S3). Approximately 38.5% of the peptides had an SA score of up to 6.0, indicating the feasibility of synthesis. All compounds had good ADMET properties.

### 2.4. Medicinal Chemistry Studies

Small molecules defined as “drug-like” need to satisfy Lipinski’s rule of five (Ro5): MW <500 Da, ≤5 H-bond donors, ≤10 H-bond acceptors, and 1−octanol/water partition coefficient (LogP) <5. Molecules that satisfy these criteria are likely to be orally bioavailable. Several studies have demonstrated that the physicochemical and structural properties of peptides are outside the traditional chemical space of approved drugs [94,95,96] based on Ro5 criteria [97]. Medicinal chemistry parameters such as MW, topological polar surface area (tPSA), LogP, fraction of sp3-hybridized carbon atoms (Fsp3), number of rotatable bonds (NRB), number of hydrogen bond acceptors (HBAs), number of hydrogen bond donors (HBDs), and number of aromatic rings (NARs) were evaluated (Table 5). 

Santos et al. analyzed peptides approved by the FDA between 2012 and 2016 to allow comparison to the Ro5 [96]. The peptides with the highest oral availability had an MW of 1200 Da and a LogP of 5–8. Furthermore, these peptides had five times more H-bond donors and acceptors than what was considered acceptable by Ro5 for small molecules [96]. High MW, tPSA, and NRB limit passive transport across cell membranes because of increased molecular size and complexation with water molecules [98,99]. HBAs and HBDs are relevant factors for cell permeability by Ro5 [100]. Our results agreed with the number of HBAs and HBDs for linear and cyclic pentapeptides and two CPP libraries [44,95]. However, the number of HBAs and HBDs in predicted peptides differed from those of clinically approved drugs [100]. The NRB and Fsp3 are used to assess molecular flexibility and complexity. The NRBs of the predicted peptides (37 to 117) exceed the maximum value for oral drugs and peptides [95,96]. The Fsp3 correlates with solubility in the aqueous phase and melting point [101]. The Fsp3 of the predicted peptides was 0.45–0.80, similar to values of orally available peptides (90th percentile = 0.79). Lipophilicity was investigated using LogP and NAR. LogP values are positively correlated with lipophilicity and thus membrane penetration. The LogP of the evaluated peptides varied from −7.387 to 0.562, consistent with values for approved peptide drugs and small molecule drugs [96,100]. The addition of an aromatic ring can significantly increase LogP [102]. Our study found that the NAR varied from 2 to 5. 

### 2.5. Prediction of Peptide Structures

After analyzing the physicochemical properties of the peptides, hydrophobicity, hydrophobic moment, GRAVY, Boman index, and ADMET parameters, fifteen BAP sequences of AMPs and CPPs with characteristics considered promising were selected for further studies. Among the 3D structures obtained, it was possible to observe the presence of a random coil, alpha helix, and a peptide sequence (NATT1.2_07) with a beta sheet structure.

**Figure 5:**
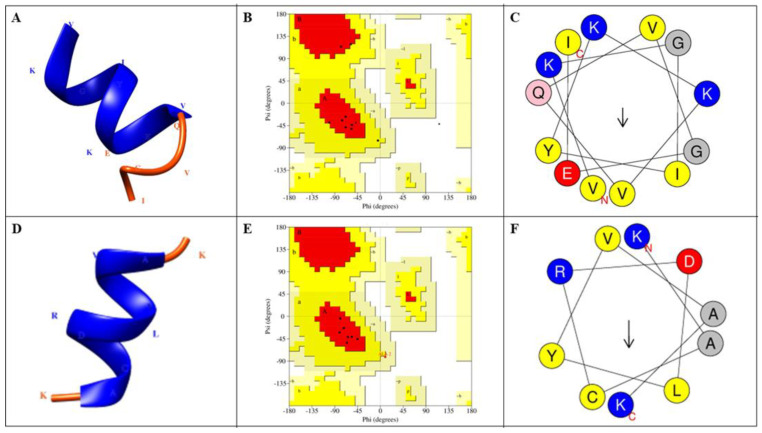
Selected PEP FOLD predicted 3D structure homology models, Ramachandran validation plots, and helical wheel projections. (**A**) NATT4_15 motif, (**B**) Ramachandran plot for the NATT4_15 motif, (**C**) NATT4_15 helical wheel projection, (**D**) NATT4_02 motif, (**E**) Ramachandran plot for the NATT4_02 motif, and (**F**) NATT4_02 helical wheel projection. NATT4_15 had nine amino acid sequences in the allowed region, whereas NATT4_02 had eight amino acids in the favorable region. These two peptides had no amino acid sequence in the unfavorable region. The graphical representations were rendered using USCF Chimera [106]. Arrows indicate the direction of the hydrophobic moment (μH).

The 3D structures were predicted using the PEP-FOLD3 web server. PEP-FOLD models for the peptides NATT1_04, NATT1.2_05, NATT1.2_06, NATT1.2_07, NATT2_06, NATT2_07, NATT2_13, NATT2_14, NATT3_03, NATT3_04, NATT4_01, NATT4_02, NATT4_06, NATT4_15, and NATTP_05 were recognized as the best with the lowest optimized potential for efficient structure prediction (sOPEP) energy (−15.1734 to −1.97158). The models with sOPEP energy of −15.1734 and −14.3622 were considered the best and are presented in Figure 5. Ramachandran plot analysis indicated that these two models had 77.8% and 87.5% of the residues in the most favorable region and 0% and 22.2% of the residues in the favorable region, respectively. In addition, the helical wheel projection of these short peptides was obtained using the Heliquest web server (Figure 5). A hydrophobic face on a helical wheel is characterized by at least five adjacent hydrophobic residues (Leu, Ile, Ala, Val, Pro, Met, Phe, Trp, or Tyr) [103].

The pH-dependent conformational equilibrium of the peptides was predicted using DispHred [104]. Khandogin [105] showed that increasing pH increased the length of the helical segments of C peptide from ribonuclease, where the difference in the relative populations of unfolded states gave rise to the pH-dependent total helix content. Our results showed that at pH 1.5 and 7.0, 75% of the peptides are in the unfolded state, with data indicating the presence of partial helices. The results provided information on the pH-dependent distribution of folded and unfolded states of the peptides. However, further in vitro studies are necessary to corroborate these data.

## 3. Materials and Methods

### 3.1. Study Design

**Figure 6:**
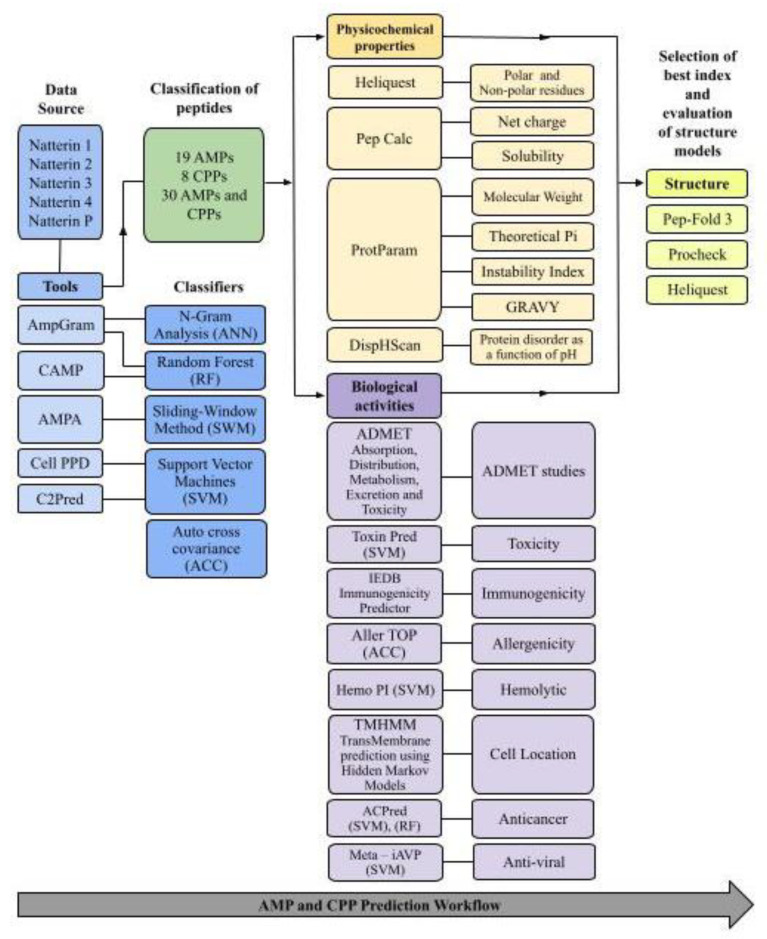
Flowchart of peptide prediction and analysis.

The current study used several in silico approaches to find and design novel and potent AMPs and CPPs as a drug delivery system. The flowchart of peptide prediction and analysis is illustrated in Figure 6.

### 3.2. Prediction of BAPs

BAPs from natterin 1 (UniProt Q66S25), natterin 2 (UniProt Q66S21), natterin 3 (UniProt Q66S17), natterin 4 (UniProt Q66S13), and natterin P (UniProt Q66S08) were predicted using bioinformatics tools. AMPs were predicted using AMPA (http://tcoffee.crg.cat/apps/ampa/do, accessed on 5 January 2022), CAMP algorithm (http://www.camp.bicnirrh.res.in, accessed on 5 January 2022) (based on primary amino acid sequences), and AmpGram (http://biongram.biotech.uni.wroc.pl/AmpGram, accessed on 5 August 2022) [107], which employs n-grams (amino acid motifs) and random forests for prediction. All sequences were in FASTA format, and antimicrobial domains were detected for the design of AMPs [108,109]. CPPs were predicted using the SVM-based web server CellPPD (https://webs.iiitd.edu.in/raghava/cellppd/index.html) and C2Pred (http://lin-group.cn/server/C2Pred, accessed both on 10 January 2022) [54,55,56]. The sequences were submitted to a protein scanning tool with the default threshold of the SVM-based prediction method [110]. 

### 3.3. Physicochemical Properties

The physicochemical parameters of peptide sequences were evaluated using different tools. MW, net charge, theoretical isoelectric point (pI), instability index, and GRAVY were estimated using ProtParam, available on the bioinformatics resource portal ExPASy of the Swiss Institute of Bioinformatics website (http://web.expasy.org/protparam, accessed on 19 October 2021). Peptide solubility and net charge at pH 7.0 were evaluated using PepCalc (https://pepcalc.com/, accessed on 24 January 2022). Hydrophobic moment (μH), hydrophobicity (H), and amino acid charge were estimated using Heliquest (https://heliquest.ipmc.cnrs.fr/cgi-bin/ComputParams.py, accessed on 24 January 2022) and the Antimicrobial Peptide Database (APD3) (https://aps.unmc.edu/prediction/predict, accessed on 25 January 2022). 

### 3.4. Evaluation of the Membrane-Binding Ability of BAPs

The Boman index and protein-binding potential were evaluated using APD3 (http://aps.unmc.edu/AP/prediction/prediction_main.php, accessed on 5 February 2022). The Boman index is the sum of solubility values for all amino acids in a peptide sequence and indicates the ability to bind to the cell membrane or other proteins [72]. The cellular localization of BAPs was assessed using the TMHMM web server (http://www.cbs.dtu.dk/services/TMHMM, accessed on 8 February 2022). TMHMM analyzes the probability of a peptide to bind to the negatively charged bacterial cell membranes.

### 3.5. Assessment of Immunogenicity, Toxicity, Allergenicity, and Anticancer and Antiviral Properties

Peptides can induce immune responses in vivo, resulting in allergic reactions. Neutralizing antibodies bind to proteins, reducing the therapeutic efficacy of these proteins [77,78]. Immunogenicity was evaluated using IEDB Immunogenicity Predictor (http://tools.iedb.org/immunogenicity, accessed on 9 February 2022) [57]. Toxicity and allergenicity were analyzed using ToxinPred (https://webs.iiitd.edu.in/raghava/toxinpred/algo.php, accessed on 10 February 2022) and AllerTop (https://www.ddg-pharmfac.net/AllerTOP, accessed on 10 February 2022) (http://ddg-pharmfac.net/AllergenFP, accessed on 11 February 2022) [61,62]. Antiviral and anticancer peptides were predicted using Meta-iAVP (http://codes.bio/meta-iavp, accessed on 12 February 2022) and ACPred (http://codes.bio/acpred, accessed on 12 February 2022), respectively [111,112]. 

### 3.6. Hemolytic Activity and Half-Life

Hemolytic activity was predicted using the SVM-based HemoPI (https://webs.iiitd.edu.in/raghava/hemopi/design.php, accessed on 12 February 2022). Peptide half-life in *E. coli* and mammalian cells was calculated using ProtParam (https://web.expasy.org/protparam, accessed on 12 February 2022). 

### 3.7. Prediction of ADMET and Medicinal Chemistry Parameters

The Simplified Molecular Input Line Entry System (SMILES) structural format of 58 peptides was obtained using PepSMI (https://www.novoprolabs.com/tools/convert-peptide-to-smiles-string, accessed on 2 April 2022). PepSMI runs an algorithm that converts raw sequences into a string of texts and unambiguously describes each atom and molecular bond in a manner amenable to machine processing. ADMET parameters, including human intestinal absorption (HIA), mutagenicity, carcinogenicity, central nervous system penetration, drug-induced liver injury (DILI), cytochrome P450 enzyme inhibition, carcinogenicity, mutagenicity, clearance, half-life, and skin sensitization, were assessed using version 420 (released on July 2021) of the ADMETlab 2.0 platform (https://admetmesh.scbdd.com/, accessed on 4 April 2022) and a comprehensive database composed of 0.25 million entries from PubChem, Online Chemical Modeling Environment (OCHEM), DrugBank, ChEMBL, Toxicity Estimation Software Tools (developed by the U.S. Environmental Protection Agency), and peer−reviewed literature [93]. Pan-assay interference compounds (PAINS) and undesirable reactive compounds were analyzed using the PAINS and Pfizer rules [113]. The ADMETlab 2.0 platform predicts the pharmacokinetic parameters based on basic information and experimental values of the respective entries. 

### 3.8. Prediction of Peptide Structure

The three-dimensional (3D) structures of predicted BAPs were analyzed using PEP-FOLD3 (https://bioserv.rpbs.univ-paris-diderot.fr/services/PEP-FOLD3/, accessed on 3 April 2022), which predicts peptide structures de novo based on primary amino acid sequences. Peptides are described as a series of fragments of four amino acids, overlapping by three, and each fragment is associated with a geometric descriptor [114]. The quality of the best models was assessed. Peptide structures were validated using PROCHECK to measure the stereochemical properties of the modeled peptide motifs [115]. Furthermore, the helical wheel diagram of peptides was defined by Schiffer Edmundson wheel modeling using Heliquest (https://heliquest.ipmc.cnrs.fr/cgi-bin/ComputParams.py, accessed on 5 April 2022) [103]. pH-dependent folded and unfolded states were predicted using SVM-based DispHred (https://ppmclab.pythonanywhere.com/DispHred, accessed on 5 August 2022) [104].

## 4. Conclusions

Fifty-seven novel and potent AMPs and CPPs derived from natterins were predicted in silico from natterin toxins. Moreover, we predicted novel peptides that had high binding membrane indexes and localization inside cells. These peptide sequences can be further evaluated for antimicrobial, cell penetration, and anticancer activity in vitro and in vivo in advance. Generally, the predicted and engineered toxin-derived AMPs and CPPs with different properties can be applied to deliver different cargoes and drug development. Overall, the present study showed that using machine learning tools in peptide research can streamline the development of targeted peptide therapies.

## Figures and Tables

**Table 1:** Predicted antimicrobial peptides (AMPs) and cell-penetrating peptides (CPPs) and their uptake efficiency.

Peptides			AMP Prediction			CPP Prediction			
Name	Sequence	AA (*n*)	CAMP	AMPA	AmpGram	C2Pred		CELL PPD	
						Prediction	Probability	Prediction	SVM Score
NATT1_01	TCKTNRIYVGKGAY	14	0	AMP	0.750	Non-CPP	0.836	Non-CPP	−0.380
NATT1_02	MRKSTVNNKQCKEVTK	16	0	AMP	0.492	CPP	0.530	Non-CPP	−0.250
NATT1_03	VNKDVIEQTM	10	0.501	-	0.047	Non-CPP	0.942	Non-CPP	−0.780
NATT1_04	DVIEQTMKDV	10	0.549	-	0.005	Non-CPP	0.912	Non-CPP	−0.640
NATT1_05	TESQSYMVTV	10	0.547	-	0.000	CPP	0.756	Non-CPP	−0.820
NATT1.2_01	RTYRGGKKTQTTTKGVYRTTQV	22	0	AMP	0.531	Non-CPP	0.524	Non-CPP	−0.350
NATT1.2_02	STNDETNLHW	10	0.524	-	0.000	Non-CPP	0.732	Non-CPP	−0.780
NATT1.2_03	CKTNRIYVGK	10	0.603	-	0.921	Non-CPP	0.657	Non-CPP	−0.100
NATT1.2_04	KTNRIYVGKG	10	0.544	-	0.561	Non-CPP	0.784	Non-CPP	−0.120
NATT1.2_05	LIRTYRGGKK	10	0.699	-	0.544	CPP	0.882	CPP	0.300
NATT1.2_06	IRTYRGGKKT	10	0.613	-	0.541	CPP	0.864	CPP	0.010
NATT1.2_07	RTYRGGKKTQ	10	0.526	-	0.413	CPP	0.537	CPP	0.000
NATT2_01	TCKTNKIYVGKGAY	14	0	AMP	0.996	Non-CPP	0.835	Non-CPP	−0.460
NATT2_02	RTYRGGKKTQTTTKGVYRTIQV	22	0	AMP	0.530	CPP	0.655	Non-CPP	−0.340
NATT2_03	TLRPKLKSKKPAK	13	0	AMP	1000	CPP	0.947	CPP	0.630
NATT2_04	TETQSYMVTV	10	0.684	-	0.000	Non-CPP	0.238	Non-CPP	−0.810
NATT2_05	ETQSYMVTVS	10	0.542	-	0.000	Non-CPP	0.238	Non-CPP	−0.710
NATT2_06	TTLRPKLKSK	10	0.505	-	0.945	CPP	0.978	CPP	0.300
NATT2_07	TLRPKLKSKK	10	0.602	-	0.991	CPP	0.952	CPP	0.540
NATT2_08	LRPKLKSKKP	10	0.565	-	0.987	CPP	0.952	CPP	0.460
NATT2_09	RPKLKSKKPA	10	0.533	-	0.975	CPP	0.929	CPP	0.290
NATT2_10	PKLKSKKPAK	10	0.638	-	1000	CPP	0.929	CPP	0.510
NATT2_11	KLKSKKPAKP	10	0.627	-	1000	CPP	0.929	CPP	0.510
NATT2_12	LKSKKPAKPA	10	0.573	-	1000	CPP	0.908	CPP	0.100
NATT2_13	KSKKPAKPAG	10	0.529	-	1000	CPP	0.682	CPP	0.200
NATT2_14	SKKPAKPAGK	10	0.556	-	1000	CPP	0.682	CPP	0.150
NATT2_15	LRPKLKSKKPAKPAGK	16	0	-	1000	CPP	0.878	CPP	0.180
NATT3_01	VYVGKNKYGLGKVHTKHE	18	0	AMP	0.996	Non-CPP	0.186	Non-CPP	−0.520
NATT3_02	MTRTYRNGQKRTTSITGTYRAIQ	23	0	AMP	0.015	CPP	0.838	Non-CPP	−0.220
NATT3_03	YVCSCGCSSG	10	0.574	-	0.577	Non-CPP	0.184	Non-CPP	−0.680
NATT3_04	CSCGCSSGFY	10	0.548	-	0.406	Non-CPP	0.204	Non-CPP	−0.650
NATT3_05	HYAYGETEKT	10	0.501	-	0.001	CPP	0.508	Non-CPP	−0.510
NATT3_06	KYGLGKVHTK	10	0.546	-	0.993	Non-CPP	0.294	Non-CPP	−0.120
NATT3_07	PPNHYCPVTM	10	0.582	-	0.949	Non-CPP	0.198	Non-CPP	−0.550
NATT3_08	PNHYCPVTMV	10	0.538	-	0.885	Non-CPP	0.246	Non-CPP	−0.410
NATT3_09	TRTYRNGQKR	10	0.531	-	0.168	CPP	0.843	CPP	0.190
NATT3_10	RTYRNGQKRT	10	0.528	-	0.166	Non-CPP	0.486	CPP	0.100
NATT4_01	LYVAKNKYGLGKL	13	0.772	-	0.989	Non-CPP	0.089	Non-CPP	−0.270
NATT4_02	KACRDLYVAK	10	0	-	0.443	Non-CPP	0.144	CPP	0.030
NATT4_03	KITNVRYNMK	10	0	-	0.045	Non-CPP	0.406	CPP	0.070
NATT4_04	IPFTGRLTRK	10	0	-	0.418	Non-CPP	0.494	CPP	0.140
NATT4_05	PFTGRLTRKY	10	0	-	0.442	CPP	0.751	CPP	0.750
NATT4_06	FTGRLTRKYS	10	0	-	0.358	CPP	0.751	CPP	0.010
NATT4_07	TGRLTRKYSN	10	0	-	0.361	CPP	0.524	CPP	0.030
NATT4_08	GRLTRKYSNG	10	0.519	-	0.406	CPP	0.746	CPP	0.040
NATT4_09	RLTRKYSNGK	10	0	-	0.412	CPP	0.830	CPP	0.160
NATT4_10	KNKYGLGKLHQS	12	0	AMP	0.989	CPP	0.604	Non-CPP	−0.160
NATT4_11	KANIPFTGRLTRK	13	0.516	-	0.449	CPP	0.702	CPP	0.050
NATT4_12	GRLTRKYSNGKVT	13	0.519	-	0.432	CPP	0.804	Non-CPP	−0.110
NATT4_13	KVTSSSVKGIYKK	13	0.601	-	0.908	Non-CPP	0.231	Non-CPP	−0.050
NATT4_14	VTSSSVKGIYKKV	13	0.508	-	0.971	Non-CPP	0.231	Non-CPP	−0.430
NATT4_15	VKGIYKKVQVGEI	13	0.746	-	0.919	Non-CPP	0.186	Non-CPP	−0.620
NATTP_01	LGQALIPRCRKMP	13	0.609	-	0.986	Non-CPP	0.468	CPP	0.150
NATTP_02	RCRKMPGVKM	10	0	-	0.634	CPP	0.767	CPP	0.010
NATTP_03	QALIPRCRKMPGV	13	0.526	-	0.990	CPP	0.547	Non-CPP	−0.090
NATTP_04	ALIPRCRKMPGVK	13	0.771	-	0.990	CPP	0.547	CPP	0.280
NATTP_05	LIPRCRKMPGVKM	13	0.645	AMP	0.893	CPP	0.563	CPP	0.050
Inference/Reference range			>0.5: AMP	-	>0.5: AMP	>0.5: CPP		SVM score >0: CPP	
			<0.5: non-AMP		<0.5: non-AMP	<0.5: non-CPP		SVM score <0: non-CPP	

**Table 2:** Amino acid characteristics of predicted bioactive peptides.

Peptides		MW (g/mol)	Polar Residues + GLY (n/%)	Uncharged Residues + GLY	Charged Residues	Non-Polar Residues (n/%)
Name	Sequence					
NATT1_01	TCKTNRIYVGKGAY	1573.83	8/57.14	THR 2, ASN 1, GLY 2	LYS 2, ARG 1,	6/42.86
NATT1_02	MRKSTVNNKQCKEVTK	1894.24	12/75.00	GLN 1, SER 1, THR 2, ASN 2, GLY 0	LYS 4, ARG 1, GLU 1	4/25.00
NATT1_03	VNKDVIEQTM	1176.35	6/60.00	GLN 1, THR 1, ASN 1, GLY 0	LYS 1, GLU 1, ASP 1,	4/40.00
NATT1_04	DVIEQTMKDV	1177.34	6/60.00	GLN 1, THR 1, GLY 0	LYS 1, GLU 1, ASP 2,	4/40.00
NATT1_05	TESQSYMVTV	1144.26	6/60.00	GLN 1, SER 2, THR 2, GLY 0	GLU 1,	4/40.00
NATT1.2_01	RTYRGGKKTQTTTKGVYRTTQV	2530.87	18/81.82	GLN 2, THR 7, GLY 3	LYS 3, ARG 3,	4/18.18
NATT1.2_02	STNDETNLHW	1216.23	8/80.00	HIS 1, SER 1, THR 2, ASN 2, GLY 0	GLU 1, ASP 1,	2/20.00
NATT1.2_03	CKTNRIYVGK	1181.42	6/60.00	THR 1, ASN 1, GLY 1	LYS 2, ARG 1,	4/40.00
NATT1.2_04	KTNRIYVGKG	1135.33	7/70.00	THR 1, ASN 1, GLY 2	LYS 2, ARG 1,	3/30.00
NATT1.2_05	LIRTYRGGKK	1191.44	7/70.00	THR 1, GLY 2	LYS 2, ARG 2,	3/30.00
NATT1.2_06	IRTYRGGKKT	1179.39	8/80.00	THR 2, GLY 2	LYS 2, ARG 2,	2/20.00
NATT1.2_07	RTYRGGKKTQ	1194.36	9/90.00	GLN 1, THR 2, GLY 2	LYS 2, ARG 2,	1/10.00
NATT2_01	TCKTNKIYVGKGAY	1545.82	8/57.14	THR 2, ASN 1, GLY 2	LYS 3,	6/42.86
NATT2_02	RTYRGGKKTQTTTKGVYRTIQV	2542.92	17/77.27	GLN 2, THR 6, GLY 3	LYS 3, ARG 3,	5/22.73
NATT2_03	TLRPKLKSKKPAK	1494.89	8/61.54	SER 1, THR 1, GLY 0	LYS 5, ARG 1,	5/38.46
NATT2_04	TETQSYMVTV	1158.29	6/60.00	GLN 1, SER 1, THR 3, GLY 0	GLU 1,	4/40.00
NATT2_05	ETQSYMVTVS	1144.26	6/60.00	GLN 1, SER 2, THR 2, GLY 0	GLU 1,	4/40.00
NATT2_06	TTLRPKLKSK	1171.45	7/70.00	SER 1, THR 2, GLY 0	LYS 3, ARG 1,	3/30.00
NATT2_07	TLRPKLKSKK	1198.52	7/70.00	SER 1, THR 1, GLY 0	LYS 4, ARG 1,	3/30.00
NATT2_08	LRPKLKSKKP	1194.53	6/60.00	SER 1, GLY 0	LYS 4, ARG 1,	4/40.00
NATT2_09	RPKLKSKKPA	1152.45	6/60.00	SER 1, GLY 0	LYS 4, ARG 1,	4/40.00
NATT2_10	PKLKSKKPAK	1124.44	6/60.00	SER 1, GLY 0	LYS 5,	4/40.00
NATT2_11	KLKSKKPAKP	1124.44	6/60.00	SER 1, GLY 0	LYS 5,	4/40.00
NATT2_12	LKSKKPAKPA	1067.34	5/50.00	SER 1, GLY 0	LYS 4,	5/50.00
NATT2_13	KSKKPAKPAG	1011.23	6/60.00	SER 1, GLY 1	LYS 4,	4/40.00
NATT2_14	SKKPAKPAGK	1011.23	6/60.00	SER 1, GLY 1	LYS 4,	4/40.00
NATT2_15	LRPKLKSKKPAKPAGK	1747.20	9/56.25	SER 1, GLY 1	LYS 6, ARG 1,	7/43.75
NATT3_01	VYVGKNKYGLGKVHTKHE	2057.38	12/66.67	HIS 2, THR 1, ASN 1, GLY 3	LYS 4, GLU 1,	6/33.33
NATT3_02	MTRTYRNGQKRTTSITGTYRAIQ	2704.06	17/73.91	GLN 2, SER 1, THR 6, ASN 1, GLY 2	LYS 1, ARG 4,	6/26.09
NATT3_03	YVCSCGCSSG	965.08	5/50.00	SER 3, GLY 2	-	5/50.00
NATT3_04	CSCGCSSGFY	1013.12	5/50.00	SER 3, GLY 2	-	5/50.00
NATT3_05	HYAYGETEKT	1198.25	7/70.00	HIS 1, THR 2, GLY 1	LYS 1, GLU 2,	3/30.00
NATT3_06	KYGLGKVHTK	1130.36	7/70.00	HIS 1, THR 1, GLY 2	LYS 3,	3/30.00
NATT3_07	PPNHYCPVTM	1158.36	3/30.00	HIS 1, THR 1, ASN 1, GLY 0	-	7/70.00
NATT3_08	PNHYCPVTMV	1160.37	3/30.00	HIS 1, THR 1, ASN 1, GLY 0	-	7/70.00
NATT3_09	TRTYRNGQKR	1279.42	9/90.00	GLN 1, THR 2, ASN 1, GLY 1	LYS 1, ARG 3,	1/10.00
NATT3_10	RTYRNGQKRT	1279.42	9/90.00	GLN 1, THR 2, ASN 1, GLY 1	LYS 1, ARG 3,	1/10.00
NATT4_01	LYVAKNKYGLGKL	1466.79	6/46.15	ASN 1, GLY 2	LYS 3,	7/53.85
NATT4_02	KACRDLYVAK	1166.4	4/40.00	GLY 0	LYS 2, ARG 1, ASP 1,	6/60.00
NATT4_03	KITNVRYNMK	1233.25	6/60.00	THR 1, ASN 2, GLY 0	LYS 2, ARG 1,	4/40.00
NATT4_04	IPFTGRLTRK	1188.44	6/60.00	THR 2, GLY 1	LYS 1, ARG 2,	4/40.00
NATT4_05	PFTGRLTRKY	1238.46	6/60.00	THR 2, GLY 1	LYS 1, ARG 2,	4/40.00
NATT4_06	FTGRLTRKYS	1228.42	7/70.00	SER 1, THR 2, GLY 1	LYS 1, ARG 2,	3/30.00
NATT4_07	TGRLTRKYSN	1195.34	8/80.00	SER 1, THR 2, ASN 1, GLY 1	LYS 1, ARG 2,	2/20.00
NATT4_08	GRLTRKYSNG	1151.29	8/80.00	SER 1, THR 1, ASN 1, GLY 2	LYS 1, ARG 2,	2/20.00
NATT4_09	RLTRKYSNGK	1222.41	8/80.00	SER 1, THR 1, ASN 1, GLY 1	LYS 2, ARG 2,	2/20.00
NATT4_10	KNKYGLGKLHQS	1372.59	9/75.00	GLN 1, HIS 1, SER 1, ASN 1, GLY 2	LYS 3,	3/25.00
NATT4_11	KANIPFTGRLTRK	1501.8	8/61.54	THR 2, ASN 1, GLY 1	LYS 2, ARG 2,	5/38.46
NATT4_12	GRLTRKYSNGKVT	1479.7	10/76.92	SER 1, THR 2, ASN 1, GLY 2	LYS 2, ARG 2,	3/23.08
NATT4_13	KVTSSSVKGIYKK	1424.7	9/69.23	SER 3, THR 1, GLY 1	LYS 4,	4/30.77
NATT4_14	VTSSSVKGIYKKV	1395.66	8/61.54	SER 3, THR 1, GLY 1	LYS 3,	5/38.46
NATT4_15	VKGIYKKVQVGEI	1460.78	7/53.85	GLN 1, GLY 2	LYS 3, GLU 1,	6/46.15
NATTP_01	LGQALIPRCRKMP	1482.87	5/38.46	GLN 1, GLY 1	LYS 1, ARG 2,	8/61.54
NATTP_02	RCRKMPGVKM	1205.56	5/50.00	GLY 1	LYS 2, ARG 2,	5/50.00
NATTP_03	QALIPRCRKMPGV	1468.84	5/38.46	GLN 1, GLY 1	LYS 1, ARG 2,	8/61.54
NATTP_04	ALIPRCRKMPGVK	1468.89	5/38.46	GLY 1	LYS 2, ARG 2,	8/61.54
NATTP_05	LIPRCRKMPGVKM	1259.0	5/38.46	GLY 1	LYS 2, ARG 2,	8/61.54

**Table 3:** Biological activities of predicted antimicrobial and cell-penetrating peptides.

Peptides		Immunogenicity	Allergenicity	Hemolysis (%)	T1/2 *Escherichia coli*	T1/2 in Mammalian (in hours)	Antiviral		Anticancer	
Name	Sequence						Prediction	Probability	Prediction	Probability
NATT1_01	TCKTNRIYVGKGAY	6082	Non-allergen	0.48	>10 h	7.2	Non-AVP	0.344	ACP	0.982
NATT1_02	MRKSTVNNKQCKEVTK	−6046	Allergen	0.49	>10 h	30	Non-AVP	0	Non-ACP	0.414
NATT1_03	VNKDVIEQTM	14,129	Non-allergen	0.49	>10 h	100	Non-AVP	0.31	ACP	0.692
NATT1_04	DVIEQTMKDV	−26,539	Allergen	0.49	>10 h	1.1	Non-AVP	0.282	ACP	0.695
NATT1_05	TESQSYMVTV	−44,274	Allergen	0.49	>10 h	7.2	Non-AVP	0.112	Non-ACP	0.639
NATT1.2_01	RTYRGGKKTQTTTKGVYRTTQV	−3531	Allergen	0.49	2 min	1	Non-AVP	0.004	ACP	0.933
NATT1.2_02	STNDETNLHW	15,897	Non-allergen	0.49	>10 h	1.9	Non-AVP	0.068	Non-ACP	0.837
NATT1.2_03	CKTNRIYVGK	23,725	Non-allergen	0.48	>10 h	1.2	Non-AVP	0.008	ACP	0.983
NATT1.2_04	KTNRIYVGKG	11,744	Non-allergen	0.48	3 min	1.3	Non-AVP	0	ACP	0.947
NATT1.2_05	LIRTYRGGKK	3716	Non-allergen	0.46	2 min	5.5	AVP	0.964	ACP	0.911
NATT1.2_06	IRTYRGGKKT	−18,382	Non-allergen	0.49	>10 h	20	AVP	0.962	ACP	0.906
NATT1.2_07	RTYRGGKKTQ	−24,544	Non-allergen	0.49	2 min	1	AVP	0.668	ACP	0.686
NATT2_01	TCKTNKIYVGKGAY	−19,958	Non-allergen	0.49	>10 h	7.2	Non-AVP	0	ACP	0.994
NATT2_02	RTYRGGKKTQTTTKGVYRTIQV	−27,354	Allergen	0.49	2 min	1	Non-AVP	0.068	ACP	0.944
NATT2_03	TLRPKLKSKKPAK	−98,576	Non-allergen	0.48	>10 h	7.2	Non-AVP	0.008	ACP	0.67
NATT2_04	TETQSYMVTV	−37,644	Allergen	0.49	>10 h	7.2	Non-AVP	0.068	Non-ACP	0.5
NATT2_05	ETQSYMVTVS	−28,293	Allergen	0.49	>10 h	1	Non-AVP	0.112	Non-ACP	0.653
NATT2_06	TTLRPKLKSK	−46,142	Non-allergen	0.49	>10 h	7.2	AVP	0.524	ACP	0.836
NATT2_07	TLRPKLKSKK	−68,378	Non-allergen	0.48	>10 h	7.2	AVP	0.616	ACP	0.703
NATT2_08	LRPKLKSKKP	−90,513	Non-allergen	0.49	2 min	5.5	AVP	0.696	Non-ACP	0.345
NATT2_09	RPKLKSKKPA	−84,374	Non-allergen	0.49	2 min	1	Non-AVP	0	Non-ACP	0.445
NATT2_10	PKLKSKKPAK	−7812	Non-allergen	0.49	ND	>20	Non-AVP	0	ACP	0.895
NATT2_11	KLKSKKPAKP	−75,989	Non-allergen	0.49	3 min	1.3	Non-AVP	0	ACP	0.894
NATT2_12	LKSKKPAKPA	−64,315	Allergen	0.49	2 min	5.5	Non-AVP	0.318	ACP	0.757
NATT2_13	KSKKPAKPAG	−4492	Allergen	0.49	3 min	1.3	AVP	0.654	ACP	0.919
NATT2_14	SKKPAKPAGK	−21,068	Allergen	0.49	10 h	1.9	AVP	0.654	ACP	0.992
NATT2_15	LRPKLKSKKPAKPAGK	−0.91	Non-allergen	0.48	2 min	5.5	Non-AVP	0	ACP	0.848
NATT3_01	VYVGKNKYGLGKVHTKHE	−59,206	Allergen	0.48	>10 h	100	Non-AVP	0.332	ACP	0.957
NATT3_02	MTRTYRNGQKRTTSITGTYRAIQ	16,556	Non-allergen	0.49	>10 h	30	Non-AVP	0.44	Non-ACP	0.444
NATT3_03	YVCSCGCSSG	−4905	Allergen	0.49	2 min	2.8	Non-AVP	0.398	ACP	0.996
NATT3_04	CSCGCSSGFY	−25,573	Non-allergen	0.49	>10 h	1.2	Non-AVP	0.104	ACP	0.998
NATT3_05	HYAYGETEKT	13,452	Allergen	0.49	>10 h	3.5	Non-AVP	0.006	ACP	0.918
NATT3_06	KYGLGKVHTK	−8.832	Allergen	0.48	3 min	1.3	Non-AVP	0.218	ACP	0.99
NATT3_07	PPNHYCPVTM	2.143	Non-allergen	0.49	ND	>20	Non-AVP	0	Non-ACP	0.873
NATT3_08	PNHYCPVTMV	875.0	Allergen	0.49	ND	>20	Non-AVP	0.126	Non-ACP	0.757
NATT3_09	TRTYRNGQKR	−13.888	Non-allergen	0.48	>10 h	7.2	Non-AVP	0.46	Non-ACP	0.672
NATT3_10	RTYRNGQKRT	−18.322	Non-allergen	0.48	2 min	1	Non-AVP	0.46	Non-ACP	0.666
NATT4_01	LYVAKNKYGLGKL	−0.45197	Allergen	0.47	2 min	5.5	AVP	0.998	ACP	0.838
NATT4_02	KACRDLYVAK	996.0	Non-allergen	0.49	3 min	1.3	AVP	0.678	ACP	0.725
NATT4_03	KITNVRYNMK	−1.485	Non-allergen	0.49	3 min	1.3	Non-AVP	0.154	ACP	0.674
NATT4_04	IPFTGRLTRK	21.302	Allergen	0.49	>10 h	20	Non-AVP	0.044	ACP	0.787
NATT4_05	PFTGRLTRKY	0.4052	Non-allergen	0.50	ND	>20	Non-AVP	0.004	ACP	0.787
NATT4_06	FTGRLTRKYS	−4.536	Non-allergen	0.49	2 min	1.1	AVP	0.542	ACP	0.9
NATT4_07	TGRLTRKYSN	−0.20894	Non-allergen	0.49	>10 h	7.2	Non-AVP	0.028	Non-ACP	0.375
NATT4_08	GRLTRKYSNG	−0.27102	Allergen	0.49	>10 h	30	AVP	0.876	ACP	0.702
NATT4_09	RLTRKYSNGK	−0.29031	Non-allergen	0.49	2 min	1	Non-AVP	0.412	ACP	0.747
NATT4_10	KNKYGLGKLHQS	−0.27934	Non-allergen	0.49	3 min	1.3	AVP	0.998	ACP	0.703
NATT4_11	KANIPFTGRLTRK	0.40878	Non-allergen	0.48	3 min	1.3	AVP	0.696	Non-ACP	0.493
NATT4_12	GRLTRKYSNGKVT	−0.42112	Non-allergen	0.49	>10 h	30	Non-AVP	0.126	Non-ACP	0.387
NATT4_13	KVTSSSVKGIYKK	−0.61671	Allergen	0.51	3 min	1.3	Non-AVP	0	ACP	0.999
NATT4_14	VTSSSVKGIYKKV	−0.69995	Allergen	0.49	>10 h	100	Non-AVP	0	ACP	0.999
NATT4_15	VKGIYKKVQVGEI	−0.22532	Allergen	0.49	>10 h	100	AVP	1	ACP	0.997
NATTP_01	LGQALIPRCRKMP	−0.012821	Allergen	0.48	2 min	5.5	Non-AVP	0	Non-ACP	0.005
NATTP_02	RCRKMPGVKM	−0.44126	Non-allergen	0.49	2 min	1	Non-AVP	0.07	Non-ACP	0.434
NATTP_03	QALIPRCRKMPGV	−0.19704	Non-allergen	0.46	10 h	0.8	Non-AVP	0.268	Non-ACP	0.015
NATTP_04	ALIPRCRKMPGVK	−0.25838	Non-allergen	0.46	>10 h	4.4	Non-AVP	0.282	Non-ACP	0.098
NATTP_05	LIPRCRKMPGVKM	−0.40468	Non-allergen	0.47	2 min	5.5	AVP	0.506	Non-ACP	0.241
Inference/Reference Range		-	SVM method	>0.5: likely hemolytic <0.5: unlikely hemolytic	ND: not determined		<0.5: low probability >0.5: high probability		<0.5: low probability >0.5: high probability	

**Table 4:** Selected ADMET properties of predicted antimicrobial and cell-penetrating peptides.

Peptides		Absorption		Distribution			Metabolism				Excretion		Toxicity				
Name	Sequence	HIA (%)	Caco-2 Permeability (cm/s)	VD (L/Kg)	BBB Penetration (%)	PPB(%)	CYP1A2-(I)	CYP1A2-(S)	CYP3A4-(I)	CYP3A4-(S)	CL (mL/min/Kg)	Half-Life	hERG Blockers	DILI Liver Injury	AMES	Carcinogenicity	Skin Sensitization
NATT1_01	TCKTNRIYVGKGAY	0.996	−7.19	0.46	0.038	22.19	0	0	0.004	0.007	0.645	0.718	0.012	0.001	0.007	0.029	0.058
NATT1_02	MRKSTVNNKQCKEVTK	0.997	−7.377	0.106	0.025	17.92	0	0	0	0.002	−0.487	0.799	0.001	0	0.043	0.041	0.06
NATT1_03	VNKDVIEQTM	0.986	−7.908	0.601	0.029	10.42	0	0	0.006	0.007	0.976	0.833	0	0.004	0.008	0.43	0.07
NATT1_04	DVIEQTMKDV	0.998	−8.074	0.657	0.018	9.97	0	0	0.006	0.007	1.171	0.914	0	0.005	0.006	0.406	0.092
NATT1_05	TESQSYMVTV	0.979	−8.024	0.428	0.042	18.47	0	0	0.008	0.009	0.919	0.88	0.001	0.02	0.005	0.074	0.035
NATT1.2_01	RTYRGGKKTQTTTKGVYRTTQV	1	−7.369	0.058	0.013	28.70	0	0	0.001	0.001	−1.957	0.892	0	0	0.001	0.005	0.005
NATT1.2_02	STNDETNLHW	0.486	−7.794	0.499	0.068	16	0	0	0.011	0.006	0.821	0.904	0.001	0.012	0.011	0.095	0.06
NATT1.2_03	CKTNRIYVGK	0.976	−7.067	0.557	0.035	9.146	0	0	0.006	0.012	1.101	0.694	0.033	0.001	0.008	0.106	0.085
NATT1.2_04	KTNRIYVGKG	0.977	−6.821	0.538	0.058	11.64	0	0	0.007	0.013	1.012	0.757	0.033	0.001	0.008	0.072	0.087
NATT1.2_05	LIRTYRGGKK	0.991	−6.768	0.569	0.048	14.74	0	0	0.012	0.013	1.067	0.782	0.036	0.001	0.007	0.079	0.116
NATT1.2_06	IRTYRGGKKT	0.997	−7.141	0.526	0.056	19.12	0	0	0.009	0.012	0.749	0.807	0.031	0.001	0.005	0.04	0.109
NATT1.2_07	RTYRGGKKTQ	0.987	−6.754	0.501	0.071	22.28	0	0	0.005	0.008	0.387	0.752	0.02	0	0.01	0.065	0.098
NATT2_01	TCKTNKIYVGKGAY	0.999	−7.282	0.475	0.027	22.20	0	0	0.006	0.007	0.745	0.812	0.004	0	0.01	0.039	0.069
NATT2_02	RTYRGGKKTQTTTKGVYRTIQV	1	−7.271	0.085	0.011	31.57	0	0	0.001	0.001	−1.65	0.887	0	0	0.001	0.008	0.006
NATT2_03	TLRPKLKSKKPAK	1	−7.301	0.232	0.014	24.84	0	0	0.001	0.005	−0.097	0.857	0.006	0	0.008	0.009	0.211
NATT2_04	TETQSYMVTV	0.99	−7.989	0.41	0.041	20.64	0	0	0.01	0.01	0.971	0.89	0	0.018	0.003	0.049	0.021
NATT2_05	ETQSYMVTVS	0.967	−7.952	0.442	0.043	18.39	0	0	0.008	0.01	0.83	0.906	0.001	0.016	0.004	0.069	0.028
NATT2_06	TTLRPKLKSK	0.991	−7.053	0.416	0.026	18.04	0	0	0.006	0.015	0.834	0.757	0.023	0.002	0.009	0.033	0.165
NATT2_07	TLRPKLKSKK	0.987	−7.063	0.349	0.043	18.41	0	0	0.005	0.013	0.956	0.742	0.029	0.002	0.047	0.031	0.244
NATT2_08	LRPKLKSKKP	0.988	−7.162	0.397	0.078	17.95	0	0.001	0.005	0.014	1.05	0.705	0.038	0.003	0.014	0.038	0.295
NATT2_09	RPKLKSKKPA	0.995	−6.974	0.403	0.069	19.56	0	0.001	0.004	0.013	0.871	0.704	0.031	0.004	0.016	0.042	0.274
NATT2_10	PKLKSKKPAK	0.999	−7.045	0.352	0.037	25.27	0	0.001	0.005	0.015	1.085	0.85	0.006	0.002	0.765	0.032	0.281
NATT2_11	KLKSKKPAKP	0.998	−7.195	0.382	0.054	20.29	0	0.001	0.005	0.016	0.761	0.833	0.008	0.003	0.214	0.054	0.341
NATT2_12	LKSKKPAKPA	0.995	−7.388	0.455	0.038	17.91	0	0.001	0.006	0.017	0.951	0.857	0.003	0.003	0.065	0.05	0.304
NATT2_13	KSKKPAKPAG	0.998	−7.252	0.480	0.038	22.41	0	0.001	0.005	0.016	0.658	0.864	0.005	0.003	0.053	0.047	0.308
NATT2_14	SKKPAKPAGK	0.998	−7.272	0.504	0.04	22.67	0	0.001	0.005	0.016	0.797	0.835	0.003	0.002	0.031	0.085	0.333
NATT2_15	LRPKLKSKKPAKPAGK	1	−7.451	0.168	0.024	23.02	0	0	0.005	0	0.248	0.821	0.006	0	0.015	0.015	0.305
NATT3_01	VYVGKNKYGLGKVHTKHE	0.999	−7.324	0.516	0.021	28.76	0	0	0.004	0.003	0.237	0.954	0.006	0.001	0.004	0.003	0.072
NATT3_02	MTRTYRNGQKRTTSITGTYRAIQ	1	−7.698	0.009	0.009	31.78	0	0	0	0.001	−2.266	0.871	0	0	0.001	0.017	0.004
NATT3_03	YVCSCGCSSG	0.727	−7.33	0.308	0.006	21.88	0	0	0.008	0.012	0.939	0.825	0.005	0.006	0.941	0.008	0.236
NATT3_04	CSCGCSSGFY	0.8	−8.059	0.391	0.009	21.41	0	0	0.01	0.011	1.039	0.821	0.011	0.007	0.84	0.03	0.289
NATT3_05	HYAYGETEKT	0.993	−7.512	0.589	0.031	36.88	0	0	0.015	0.008	1.16	0.956	0.006	0.007	0.002	0.038	0.053
NATT3_06	KYGLGKVHTK	0.994	−6.868	0.571	0.062	17.77	0	0.003	0.015	0.016	1.256	0.928	0.044	0.002	0.008	0.007	0.136
NATT3_07	PPNHYCPVTM	0.995	−6.867	0.475	0.029	30.59	0	0.006	0.009	0.018	1.412	0.853	0.006	0.833	0.006	0.033	0.077
NATT3_08	PNHYCPVTMV	0.983	−6.877	0.458	0.033	29.24	0	0.005	0.01	0.017	1.429	0.844	0.006	0.899	0.763	0.025	0.05
NATT3_09	TRTYRNGQKR	0.889	−6.91	0.447	0.078	19.44	0	0	0.003	0.005	0.195	0.685	0.011	0	0.016	0.125	0.088
NATT3_10	RTYRNGQKRT	0.91	−6.544	0.439	0.075	19.89	0	0	0.003	0.005	0.128	0.688	0.011	0	0.012	0.122	0.076
NATT4_01	LYVAKNKYGLGKL	0.989	−7.356	0.623	0.024	18.21	0	0	0.056	0.001	0.578	0.864	0.022	0.001	0.007	0.08	0.132
NATT4_02	KACRDLYVAK	0.986	−7.455	0.584	0.039	9.261	0	0	0.003	0	1.107	0.79	0.031	0.008	0.064	0.069	0.162
NATT4_03	KITNVRYNMK	0.95	−6.477	0.549	0.04	11.28	0	0	0.002	0	0.986	0.643	0.017	0.002	0.007	0.261	0.069
NATT4_04	IPFTGRLTRK	0.998	−7.063	0.47	0.028	16.47	0	0	0.006	0	1.218	0.747	0.03	0.004	0.004	0.4	0.088
NATT4_05	PFTGRLTRKY	0.998	−6.943	0.488	0.024	26.01	0	0	0.003	0	1.152	0.761	0.041	0.003	0.005	0.027	0.055
NATT4_06	FTGRLTRKYS	0.984	−7.18	0.426	0.042	16.19	0	0	0.004	0	0.879	0.782	0.029	0.002	0.006	0.028	0.058
NATT4_07	TGRLTRKYSN	0.923	−7.407	0.452	0.069	16.24	0	0	0	0	0.624	0.698	0.017	0.001	0.011	0.054	0.097
NATT4_08	GRLTRKYSNG	0.877	−7.430	0.5	0.056	18.76	0	0	0	0	0.6	0.766	0.022	0.001	0.018	0.048	0.109
NATT4_09	RLTRKYSNGK	0.921	−7.018	0.482	0.055	17.18	0	0	0	0	0.638	0.722	0.022	0.001	0.017	0.104	0.124
NATT4_10	KNKYGLGKLHQS	0.832	−6.901	0.517	0.056	20.70	0	0	0.012	0	0.830	0.873	0.022	0	0.033	0.027	0.213
NATT4_11	KANIPFTGRLTRK	0.999	−6.613	0.425	0.026	20.34	0	0	0.001	0	0.557	0.727	0.009	0.001	0.004	0.058	0.066
NATT4_12	GRLTRKYSNGKVT	0.991	−7.574	0.384	0.041	22.11	0	0	0	0	0.113	0.798	0.008	0	0.006	0.036	0.068
NATT4_13	KVTSSSVKGIYKK	1	−7.250	0.363	0.02	18.23	0	0	0.003	0	0.463	0.918	0.002	0.001	0.009	0.042	0.103
NATT4_14	VTSSSVKGIYKKV	1	−7.499	0.417	0.025	16.82	0	0	0.005	0.001	0.544	0.912	0.002	0.002	0.004	0.038	0.065
NATT4_15	VKGIYKKVQVGEI	0.999	−7.261	0.592	0.019	20.76	0	0	0.008	0.001	0.712	0.912	0.003	0.001	0.003	0.097	0.085
NATTP_01	LGQALIPRCRKMP	0.987	−6.502	0.49	0.013	19.82	0	0	0.002	0	1.006	0.578	0.012	0.003	0.01	0.037	0.162
NATTP_02	RCRKMPGVKM	0.985	−6.729	0.483	0.036	15.67	0	0.001	0.001	0	1.084	0.773	0.033	0.003	0.036	0.036	0.178
NATTP_03	QALIPRCRKMPGV	0.993	−6.490	0.483	0.014	19.08	0	0	0.001	0	0.974	0.606	0.01	0.005	0.009	0.034	0.156
NATTP_04	ALIPRCRKMPGVK	0.995	−6.759	0.488	0.011	20.18	0	0	0.002	0	1.078	0.7	0.015	0.004	0.011	0.033	0.159
NATTP_05	LIPRCRKMPGVKM	0.995	−6.721	0.467	0.009	22.53	0	0	0.004	0	1.119	0.742	0.014	0.003	0.013	0.038	0.153
Inference/Reference Range		HIA > 0.3: HIA positive HIA < 0.3: HIA negative	Optimal: higher than −5.15	Optimal: 0.04–20 L/Kg	≥0.1: BBB positive and <0.1: BBB negative	PPB < 90%: optimal PPB > 90%: low therapeutic index	>0.5: inhibitor <0.5: non inhibitor	>0.5: substrate <0.5: non substrate	>0.5: inhibitor <0.5: non-inhibitor	>0.5: substrate <0.5: non-substrate	High: >15 mL/min/kg Moderate: 5–15 mL/min/kg Low: <5 mL/min/kg	Long half-life: >3 h Short half-life: <3 h	>0.5: blocker <0.5: non-blocker	>0.5: hepatotoxic <0.5: non-hepatotoxic	>0.5: positive <0.5: negative	>0.5: carcinogen <0.5: non-carcinogen	>0.5: sensitizer <0.5: non-sensitizer

**Table 5:** Chemical spaces of predicted peptides and marketed drugs (modified from Oliveira et al., 2021).

	Oral Drugs		Peptides			
Molecular Properties	Lipinski, 2001 and Veber, 2002	Doak et al., 2014	Santos et al., 2016 *	Diaz-Eufracio et al., 2018 **	De Oliveira et al., 2021 #	Our Study
MW	≤500	≤1.000	≤700	27.03 ≤ MW ≤ 5036.65	331.48 ≤ MW ≤ 3750.51	965.08 ≤ MW ≤ 2704.06
LogP	≤5	−2 ≤ LogP ≤ 10	≤7.5	−17.87 ≤ LogP ≤39.89	−42.12 ≤ LogP ≤ 2.97	−7.387 ≤ LogP ≤ 0.562
tPSA	≤140	≤250	≤200	≤2064.83	101.29 ≤ tPSA ≤1782.83	405.88 ≤ tPSA ≤ 1288.48
Fsp^3^	−	−	≤0.55	−	0.37 ≤ Fsp^3^ ≤ 0.84	0.45 ≤ Fsp^3^ ≤ 0.80
NRB	≤10	≤20	≤20	≤209	9 ≤ NRB ≤ 137	37 ≤ NRB ≤ 117
HBA	≤10	≤15	≤10	≤71	5 ≤ HBA ≤ 55	25 ≤ HBA ≤ 75
NAR	−	−	−	−	≤10	≤5

Notes: * Investigated orally active peptides; ** investigated linear and cyclic pentapeptides; # investigated cell-penetrating peptides.

## Data Availability

Data is contained within the article and supplementary material.

## Supplementary Materials

The following supporting information can be downloaded at: https://www.mdpi.com/article/10.3390/ph15091141/s1, Supplementary Figure S1. Multiple sequence alignment analysis. Natterins from Thalassophryne nattereri venom were aligned using ClustalW (UNIT 2.3 Multiple Sequence Alignment Using ClustalW and ClustalX, 2003). Accession numbers: SP|Q66S25| Natterin 1; SP|Q66S21| Natterin 2; SP|Q66S17| Natterin 3; SP|Q66S13| Natterin 4; SP|Q66S08| Natterin P. *Fully conserved residues; conservation of strongly similar groups (>0.5 in the Gonnet PAM 250 matrix); conservation of weakly similar groups (≤0.5 in the Gonnet PAM 250 matrix) [116]. Table S1. Physicochemical proprieties of predicted antimicrobial and cell-penetrating peptides. Table S2. Membrane-binding potential and cellular localization of predicted antimicrobial and cell-penetrating peptides. Table S3. Prediction of medicinal chemistry proprieties of peptides. Reference [116] are cited in the Supplementary Materials.
