# Supplementary material for: In Silico Prediction of Anti-Infective and Cell-Penetrating Peptides from Thalassophryne nattereri Natterin Toxins
Source: Pharmaceuticals (Basel). 2022 Sep 13;15(9):1141. doi: 10.3390/ph15091141 (PMC9501638; doi:10.3390/ph15091141)
Supplement: Supplementary file 1 [file pharmaceuticals-15-01141-s001.zip › Figure S1.pdf]

```
SP|Q66S25|NATT1_THANI MIPSVLLVTLLLLSWTSAEKDLK------------------------------------- 23
SP|Q66S21|NATT2_THANI MNLSVLLVTLLLLSWTSAEKDLK------------------------------------- 23
SP|Q66S17|NATT3_THANI MKLSVLVVTLLAVSWTSAQPETFSIQ----T-------------------KEANMNPEPA 37
SP|Q66S13|NATT4_THANI MKLLVLLVTLLVLSWTSAEDVGDQEILQQHNEDNNHKSELGEAAPQRTDNETSQLGQETP 60
SP|Q66S08|NATTP_THANI MKLLVLLVTLLVLSWTSAEDLGDQEILE-NNEDNNHESELGEPAAQHTDDETSQLGQALI 59
                      *   **:**** :*****:

SP|Q66S25|NATT1_THANI -VRVAR--STNDETNLHWVKCGG--SVPDGAVSIQNTYVSPARTEYVCKSNCEAGYYSTK 78
SP|Q66S21|NATT2_THANI -VRVAR--STNDETNLHWVKCGG--SVPDGAVSIRNTYVSPARTEYVCKCFCQAGYYSTK 78
SP|Q66S17|NATT3_THANI NIRVAR--SSSAQSNLQWNYWDGQGAVPDGAVSIWNGEE--KRTDYVCSCGCSSGFYSTK 93
SP|Q66S13|NATT4_THANI TIRVARAYEFSSKSNLEWVRWNG--HIPSNAVKISNTYV--GREDYVCRVGCEAGYYTPK 116
SP|Q66S08|NATTP_THANI PRCRKMPGVKMC------------------------------------------------ 71
                       :****  . . ::**.*   .*   :*..**.* *      * :***   *.:*:*: *

SP|Q66S25|NATT1_THANI D-SKCHYPFGRVEQT-TSVCEILVNRDNFELLEWKEGYAGSLPANAVSTCKTNRIYVGKG 136
SP|Q66S21|NATT2_THANI D-SKCHYPYGTKEMATSTNCYILVNRDNFELLEWKDGYAGSVPDNAVSTCKTNKIYVGKG 137
SP|Q66S17|NATT3_THANI TGANCHYAYGETEKT-CSGFSILVNRDNFENLEWKGGSDGSVPKNAVEVCE--KVYVGKN 150
SP|Q66S13|NATT4_THANI KGPSCFYPYGFTEQH-SKMFHILVNRDNFEILEWKWKTGGEVPENAVKACR--DLYVAKN 173
SP|Q66S08|NATTP_THANI ------------------------------------------------------------
                          .*.* :*  *    .   ********* ****    *.:* ***..*.   :**.*.

SP|Q66S25|NATT1_THANI AYGLGKIEPAHHCLYYGWNGAETWTKTYQALTVNKDVIEQTMKDVKYQTEGVTVIQGKPE 196
SP|Q66S21|NATT2_THANI AYGLGKIEPANHCLYYVWDGAETWTKTYQALTMNKDVIEQAMKDVKYQTEGVTVIKGKPE 197
SP|Q66S17|NATT3_THANI KYGLGKVHTKHEALFLPWHGEEHWYKDYEVLTVNDDVVKQELTQVNYKLDAAHPIKNPPE 210
SP|Q66S13|NATT4_THANI KYGLGKLHQSHHVFYLPWKGTEYKYNEYYVLNVNMDVVEQKITNVRYNMKGVEVHKDKPE 233
SP|Q66S08|NATTP_THANI ------------------------------------------------------------
                       *****:.  :. ::  *.* *   : * .*.:* **::* :.:*.*: ...   :. **

SP|Q66S25|NATT1_THANI VMRKSTVNNKQCKEVTKTVTLSKDISTEERWDVTNSVTFGVTTTVTAGIPDVASASLAVS 256
SP|Q66S21|NATT2_THANI VMRRSTVNNQHCKEVTKTVTLTKDISTDERWDVTNSVTFGVTTTVTAGIPDVSSASLEIS 257
SP|Q66S17|NATT3_THANI TLRRSSASNSQCRPITKTVALEKAIQTEQSWDVTSTVTFGVESSITAGIPDIASATVSVS 270
SP|Q66S13|NATT4_THANI TLRSTSVKNYQCREATKQVTLEKSTETSQSWDVSNSITLGVSTEVSAGIPNIADVSVAVS 293
SP|Q66S08|NATTP_THANI ------------------------------------------------------------
                      .:* ::..* :*:  ** *:* *  .*.: ***:.::*:** : ::****:::..:: :*

SP|Q66S25|NATT1_THANI MEARRDFAHGASKTESQSYMVTVSVPVPPKQSCTVSMVAQVNKADVPFTATLIRTYRGGK 316
SP|Q66S21|NATT2_THANI MQATMDFAHGASKTETQSYMVTVSVPVPPKQSCTVSMVAQVNKADIPFTATLIRTYRGGK 317
SP|Q66S17|NATT3_THANI VETSLSVSLGSTTTKTTTHTVSVIVTVPPNHYCPVTMVATKYTADIPFTGKMTRTYRNGQ 330
SP|Q66S13|NATT4_THANI AETSVEISHGTSKTESTSHSLSVSATIPPNSSCSITMEGCTFKANIPFTGRLTRKYSNGK 353
SP|Q66S08|NATTP_THANI ------------------------------------------------------------
                       ::  ..: *::.*:: :: ::* . :**:  * ::* .   .*::***. : *.* .*:

SP|Q66S25|NATT1_THANI KTQTTTKGVYRTTQVAETHADVEQCTIIGDEKDCPKASK--------------------- 355
SP|Q66S21|NATT2_THANI KTQTTTKGVYRTIQVAETHADVEQCTIIGDAKDCPNASSTITTLRPKLKSKKPAKPAGK 376
SP|Q66S17|NATT3_THANI KRTTSITGTYRAIQVGEIRADVQRCSEIAGAKPC-------------------------- 364
SP|Q66S13|NATT4_THANI VTSSSVKGIYKKVQVGEIQAVLHRCDKIADAKPC-------------------------- 387
SP|Q66S08|NATTP_THANI ------------------------------------------------------------
                         :: .* *:  **.* :* :.:*  *.. * *
```

Figure S1. Multiple sequence alignment analysis. Natterins from Thalassophryne nattereri venom were aligned using ClustalW (UNIT 2.3 Multiple Sequence Alignment Using ClustalW and ClustalX, 2003). Accession numbers: SP|Q66S25| Natterin 1; SP|Q66S21| Natterin 2; SP|Q66S17| Natterin 3; SP|Q66S13| Natterin 4; SP|Q66S08| Natterin P. *Fully conserved residues; conservation of strongly similar groups (>0.5 in the Gonnet PAM 250 matrix); conservation of weakly similar groups (≤0.5 in the Gonnet PAM 250 matrix) [116].
